# Characterization of IGF-II Isoforms in Binge Eating Disorder and Its Group Psychological Treatment

**DOI:** 10.1371/journal.pone.0083019

**Published:** 2013-12-30

**Authors:** Giorgio Tasca, Julien Yockell Lelievre, Qing Qiu, Kerri Ritchie, John Little, Anne Trinneer, Ann Barber, Livia Chyurlia, Hany Bissada, Andreé Gruslin

**Affiliations:** 1 Psychology Department, The Ottawa Hospital, University of Ottawa, Ottawa, Ontario, Canada; 2 Department of Cellular and Molecular Medicine, The Ottawa Hospital, University of Ottawa, Ottawa, Ontario, Canada; 3 Dept Psychology, The Ottawa Hospital, Ottawa, Ontario, Canada; 4 Department of Epidemiology and Community Medicine, University of Ottawa, Ottawa, Ontario, Canada; 5 Department of Obstetrics, Gynaecology and Newborn Care, The Ottawa Hospital, University of Ottawa, Ottawa, Ontario, Canada; 6 Ottawa Hospital Research Institute (OHRI), Ottawa, Ontario, Canada; 7 Children's Hospital of Eastern Ontario, Ottawa, Ontario, Canada; 8 Department of Psychiatry, The Ottawa Hospital, University of Ottawa, Ottawa, Ontario, Canada; Kaohsiung Chang Gung Memorial Hospital, Taiwan

## Abstract

**Intro:**

Binge eating disorder (BED) affects 3.5% of the population and is characterized by binge eating for at least 2 days a week for 6 months. Treatment options include cognitive behavioral therapy, interpersonal psychotherapy, and pharmacotherapy which are associated with varied success.

Little is known about the biology of BED. Since there is evidence that the insulin like growth factor system is implicated in regulation of body weight, insulin sensitivity and feeding behavior, we speculated it may be involved in BED.

**Methods:**

A cross-sectional comparison was made between three groups of women: overweight with BED, overweight without BED and normal weight without BED. Women were assigned to Group Psychodynamic Interpersonal Psychotherapy. Blood was collected before therapy, at completion and at 6months follow up for evaluation of IGF-II using Western blot.

**Results:**

97 overweight women with BED contributed to the cross-sectional comparison. The two control groups comprised 53 overweight women without BED, and 50 age matched normal weight women without BED. Obese women had significantly lower Big IGF-II than normal weight women, *p* = .028; Overweight women with BED had higher Mature IGF-II than normal weight women, *p*<.05.

Big IGF-II showed a significant decreasing slope from pre- to post- to six months post-group psychological treatment, unrelated to changes in BMI (*p* = .008).

**Conclusion:**

Levels of IGF-II isoforms differed significantly between overweight and normal weight women. Overweight women with BED display abnormal levels of circulating IGF-II isoforms. BED is characterized by elevated mature IGF-II, an isoform shown to carry significant bioactivity. This finding is not related to BMI or to changes in body weight. The results also provide preliminary evidence that BIG IGF-II is sensitive to change due to group psychological treatment. We suggest that abnormalities in IGF-II processing may be involved in the neurobiology of BED.

## Introduction

Binge eating disorder (BED) is relatively common, affecting up to 3.5% of the general population [1]. It is characterized by episodes of binge eating at a frequency of at least 2 days a week for 6 months [2]. These episodes consist of eating, in a discrete period of time (e.g., within any 2 hour period), an amount of food that is definitely larger than most people would eat in a similar period of time under similar circumstances. In addition, there is a sense of lack of control over eating during the episode (e.g., a feeling that one cannot stop eating or control what or how much one is eating) [2]. Treatment options have been proposed and include cognitive behavioral therapy, interpersonal psychotherapy, and pharmacotherapy [3]. Common comorbidities of BED include depression and obesity [4], [5].

The diversity of therapeutic options and the heterogeneity of their respective success lies partly in the fact that the mechanism(s) underlying the development of BED is (are) unclear. Recently, the neurobiology of BED and other eating disorders has started to be examined in more detail in the hope to understand the pathophysiological mechanisms behind these disorders and to improve treatment options. As such, dopamine, opioids, ghrelin and serotonin have been suggested to play a role in BED although further studies are required to investigate these possibilities [6].

Little is known about the physiology of body weight control. However, in this context, the IGF system appears to have clinical implications. The IGF system is composed of two growth factors (IGF-I and IGF-II], six binding proteins (IGFBP-1-6) which mediate growth factor availability, and four receptors. The insulin-like growth factor (IGF) system plays an important role in cellular proliferation, differentiation and growth, as well as lipid metabolism and body weight regulation [7]. During adult life, IGF-II is expressed in many tissues and is regulated at the gene and protein level. Human IGF-II is initially synthesized as a pre-pro-IGF-II peptide which upon removal of a 24-amino acid signal peptide at the N-terminal results in the generation of pro-IGF-II. ProIGF-II is then cleaved, generating Big IGF-II (1–104), a peptide with 104 amino acids [8]. Big IGF-II (1–104) is subsequently endoproteolyzed, resulting in mature IGF-II, a 7.5 kDa peptide containing 67 amino acids [9]. Our group has previously shown that these various isoforms differ functionally and that alterations in IGF-II processing can be associated with abnormal growth, at least in the fetus [9].

In human adults, the relationship between IGF-II and body weight is supported by several studies in which low levels of “circulating IGF-II” (which consists of all the circulating isoforms) has been found to be associated with an increased risk of weight gain and obesity [7] which remained true in the presence of type II diabetes [10]. Obesity itself (a common co-morbidity in BED) has been shown to be associated with up regulation in IGF-II/mannose -6-phosphate receptor and circulating concentrations of both this receptor and IGF-II were found to be correlated with BMI [11]. Furthermore, relatively higher IGF-II levels were associated with a reduced risk of gaining weight, which further supported the role of IGF-II in body weight regulation [7]. This may be indicative of a central acting role of IGF-II in feeding behavior. Only one study to date has examined IGF-II processing in obesity and weight loss. Results showed that pro-IGF-II was decreased in obese subjects whereas mature IGF-II was increased, suggesting a possible relationship between nutritional status and pro-IGF-II [12]. To date there is no information on IGF-II expression/processing and BED.

In addition to weight regulation, other factors may support a role for IGF-II dysregulation in BED, for instance, depression and insulin resistance. Others have reported that depression severity itself is negatively correlated with serotonin levels [13] and insulin resistance is known to be associated with low serotonin levels. In addition, treatment with serotonin selective reuptake inhibitors results not only in increases in serotonin, but also decreases in IGF II expression in both the hypothalamus and the pituitary in the rat model [14]. The complex IGF-II and serotonin systems may indeed interrelate, raising the possibility that individuals with BED who are at increased risk of both depression and insulin resistance may display low serotonin levels, which in other systems have been shown to be associated with increased IGF –II expression [14]. In addition, as psychological or pharmacological therapy targets depression and body weight with expected increases in serotonin, it is possible that these abnormal levels return to near baseline following treatment.

Taken together, these data suggest that nutritional status, depressed mood, insulin sensitivity and perhaps feeding behavior may be associated with alterations in IGF II expression and processing. The associations between IGF-II expression and body weight, the possible actions of IGF-II on the hypothalamus (satiety center), and finally our previous demonstration of the various physiological activities of different IGF-II isoforms (mature being more biologically potent) led us to postulate that women with BED, independently of the presence of obesity would display abnormal processing of IGF-II when compared with healthy controls. In addition, as part of an exploratory investigation, we assessed if IGF-II changed as a result of group psychological treatment that targeted binge eating and depression [15], [16].

## Methods

### Ethics Statement

This research project was approved by OHREB (Ottawa Hospital Research Ethics Board). All participants signed informed consent forms, prior to enrollment into the study. Once enrolled, participants were assigned a unique study code, which allowed for total anonymity and confidentiality throughout the study.

### Study design and Participants

We undertook a cross-sectional comparison between three groups: (1) overweight (body mass index (BMI = />27 kg/m^2^) women with BED who responded to an invitation or advertisement to participate in a trial of group psychological treatment for BED; (2) age and weight matched overweight women without BED; and (3) age matched normal weight (BMI>20 and BMI<27) women without BED. Participant in the two BED groups of women without BED were matched to the BED sample on age, so that there were equal proportions of individuals in each decade (i.e. 18–20 years, 21–30 years, 31–40 years, etc.). Participants without BED in the overweight group were also matched to the BED sample on BMI, so that there were equal proportions in each weight class (i.e. BMI = 27–29.99, BMI = 30–34.99, BMI = 35–39.99, and BMI>40). These three groups of women were asked to provide data at one time point for the cross sectional comparisons. In addition, we compared data from the sub-sample of women with BED (a) before treatment (i.e. week 0); (b) post-group psychological treatment (i.e. week 16); and (c) six months post treatment (i.e. week 40). Therefore whereas the control group provided one measurement, the BED group was sampled three times as described above.

Exclusion criteria for all participants included: inability to speak or read English, current or past inappropriate compensatory behaviors (e.g. vomiting), drug or alcohol abuse in the past six months, a diagnosis of bipolar disorder or a psychotic disorder, pregnancy, and a diagnosis of diabetes as well as a past or current history of cancer. In addition, BED participants could not be enrolled or plan to enroll in a weight loss program for one year, could not plan to become pregnant in the next year, and could not be taking medication that would affect weight during the psychological treatment. Control condition participants could not have a current or past eating disorder including BED.

### Procedure

BED participants were women referred from a tertiary care eating disorders program or respondents to community newspaper advertisements for treatment of binge eating. A research coordinator screened participants by phone for exclusion criteria, described the nature of the study, and assessed for the presence of BED using the Structured Clinical Interview for *DSM-IV* Axis I Disorders [17]. The treatment study's main purpose was to assess if creating homogeneous therapy groups based on level of attachment anxiety (defined below) might improve group therapy outcomes. As such, based on their scores on an attachment anxiety scale, participants were assigned to therapy groups. For the purpose of this study, all individuals were assessed at pre-treatment, including having their blood drawn, and then received 16 weeks of group therapy. The group therapy was Group Psychodynamic Interpersonal Psychotherapy [15] which showed positive outcomes for BED in a previous randomized controlled trial [16]. In the treatment trial from which this BED study sample was taken, GPIP was effective in reducing frequency of binge eating and depressive symptoms from pre-treatment up to one year post treatment [18]. Each of six therapists crossed over conditions, so that they each led one group in the high attachment anxiety and one group in the low attachment anxiety condition. Both therapy group conditions received the same treatment. Therapists were blind to their group's study condition. Participants were assessed at pre-, post-, and six months post-treatment with the same measures, including blood draws.

Participants in the comparison groups were individuals who responded to advertisements in local newspapers to participate in a study on women's health. These participants were screened over the phone for BED and exclusion criteria by a research coordinator, and then invited for an in-person interview and assessment.

All of these participants were weighed, completed questionnaires, and peripheral blood was drawn. Approximately 10ccs of whole blood was obtained by peripheral venipuncture. Samples were centrifuged and sera collected and stored at −80C for later batch analysis. All participants provided informed consent.

### Measures

**Attachment Anxiety:** In the current study, participants with a score of at least 3.59 on the Attachment Style Questionnaire Need for Approval scale [19]were assigned to the high attachment anxiety condition, and those with a score below 3.59 were assigned to the low attachment anxiety condition. The ASQ is a self-administered self report questionnaire. The cut-off of 3.59 was based on Tasca, and colleagues' [16] finding that the interaction between Need for Approval scores and treatment type predicted change in post-treatment days binged. Mean inter item correlation for the Need for Approval scale was adequate at 0.34.

**Weight and Height:** Participants were weighed on the same standard scale (Tanita Body Composition Analyser, BC-418) at pre-treatment, post-treatment and six months follow-up. Height was measured with the same measuring tape on each occasion.

**Diagnoses:** The research version of the Structured Clinical Interview for *DSM-IV* Axis I Disorders [17]was used to diagnose BED, and mood and anxiety disorders. The SCID is a clinician-administered diagnostic interview that assesses frequently diagnosed Axis I *DSM-IV* disorders in adults, with a module for BED in the research version. In the current study, inter-rater reliability with Cohen's kappa indicated very good concordance between independent raters for current mood disorders, *k* = .82, and for current anxiety disorders, *k* = .83. Concordance between two raters for number of binge episodes to define a BED diagnosis by intra class correlation was ρ = .98.

### Determination of IGF-II profile by Western blot analysis

The IGF-II profile was determined by a sensitive Western blot analysis as described in our previous study [20]. This assay allows us to determine IGF-II concentrations in samples at the pg level and to differentiate between the pro, “big” and mature IGF-II isoforms with 0.5 *u*l of sera. Briefly, aliquots of 0.5 *u*l sample diluted with non-reducing loading buffer to 20 *u*l were subjected to electrophoresis with 10% Tricine SDS-PAGE. The same internal control (40 randomly selected pooled samples) was loaded on every gel. The separated proteins were electro-transferred to a nitrocellulose membrane (Bio-Rad, Canada). The membrane was treated with Miser antibody extender solution NC (Pierce, Rockford, IL) and blocked with 5% non-fat dry milk. Detection was performed for IGF-II with mouse anti-rat IGF-II monoclonal antibody (clone S1F2, Upstate, Lake Placid, NY) and Goat Anti-Mouse IgG (H+L)-HRP Conjugate (Bio-Rad, Canada) which we have reported before as being suitable for determination of IGF-II in humans [9]. Bands in all blots were visualized with ECL reagents or advanced ECL reagents (GE Healthcare, Buckinghamshire, UK) using a GeneGnome chemiluminescence imaging system (Syngene). Their relative contents were then densitometrically quantified with Genetools analysis software (Syngene) and normalized by the internal control. All the blots used in the quantification process were only ever probed once thus eliminating the potential of artifacts from a previous detection.

## Data Analysis Plan

To test differences between three groups (overweight women with BED at pre-treatment, overweight women without BED, and normal weight women without BED) on PRO, BIG, and Mature IGF-II, we conducted a one-way multivariate analysis of variance (MANOVA). Tukey's post hoc test was used to assess specific between-group differences on each variable that showed a between-groups effect. We also tested differences between overweight women (with and without BED) and normal weight women without BED using a two-group one-way MANOVA. To evaluate change in each IGF-II variable from pre- to post- to six months post-treatment in the women with BED who received group psychological treatment, we conducted a longitudinal analysis with multilevel modeling [21]. Further, to test if change in BMI was associated with change in IGF-II variables we conducted 2 level longitudinal multilevel modeling (MLM) in which repeated measurements were nested within individuals and BMI was a time varying covariate [22]. MLM has numerous advantages over repeating ANOVA including not being susceptible to common statistical violations, and not requiring complete data for each individual in order to estimate parameters [23]. That is, with MLM one does not have to impute missing values or listwise delete cases because the maximum likelihood estimation uses all available data to estimate reliable parameters for each individual even with missing data as long as the data are missing at random. To assess if the data were missing at random, we used a pattern mixture modeling approach in which a missingness pattern characterized by a case with any missing data was assessed [24]. To assess for dependence in the grouped data of BED participants we calculated the intra class correlation coefficient (ICC) for slopes from a 3-level longitudinal MLM in which individuals were nested within groups [25]. In all the MLM, baseline values of the dependent variable and group therapy condition were controlled, the linear growth parameter was fixed, and all covariates were grand mean centered. Correlational and multivariate tests were conducted using SPSS v.20. MLM was conducted with the Hierarchical Linear Modeling (HLM 6.08) software.

## Results

Of 230 individuals with BED who originally contacted us to participate in the treatment trial, 93 were ineligible, 29 declined an in-person assessment, and 11 declined treatment and provided no data, resulting in 97 overweight women with BED contributing to the cross-sectional comparison. Sixty-four of these women provided data at post-group psychological treatment, and 56 provided data at six months post treatment. Initially, 111 participants were recruited for the overweight and normal weight comparison groups, but two reported a history of binge eating, 3 were ineligible, 2 withdrew consent, and 1 did not fit the matching criteria for age, leaving 103 women. Thus, the two control groups comprised 53 age and weight matched overweight women without BED, and 50 age matched normal weight (BMI>20 and BMI<27) women without BED. Socio-demographic data on the three groups are presented in Table 1.

**Table 1:** Demographic data on the three samples.

Demographic	BED (*n* = 97)	Overweight (*n* = 53)	Normal Weight(*n* = 50)
Mean Age (*SD*)	44.38 (11.86)	46.02 (12.19)	44.53 (12.15)
Mean BMI (*SD*)	38.22 (7.20)	36.77 (6.41)	23.20 (2.02)
White (%)	89.8	88.7	87.8
Married or Cohabitating (%)	48.1	71.7	67.3
Employed Full- or Part-time (%)	86.9	83.0	93.9
Completed University/College (%)	63.5	67.8	89.6
Median Family Income (Thousands)	60–69	>80	>80

Note: BED = binge eating disorder. *SD* = standard deviation. Family income in Canadian dollars in which $1 Canadian = $0.99 US.

Preliminary analyses indicated that the variables met data analytic assumptions including normality at each time point and no univariate or multivariate outliers. Using a pattern mixture model [24] we determined that in each case the IGF-II data were missing at random, indicating that the MLM analyses with all available data resulted in reliable parameters. The ICC indicated that each dependent variable showed ignorable dependence in the grouped data (all ICC<.02), and so we proceeded to use two level models for all analyses.

We first correlated each IGF-II variable with BMI. For the total sample, *n* = 200, BMI was significantly correlated with Mature IGF-II (*r* = .21, *p*<.003) and with Big IGF-II (*r* = −.18, *p* = .01), but not significantly correlated with Pro IGF-II (*r* = −.02, *p*>.05).

We then conducted a MANOVA to assess if those who were overweight (i.e., from both the BED and non-BED sample, *n* = 160), differed from the normal weight women, *n* = 50, on the IGF-II variables. The multivariate test was significant, *p*<.001. Follow-up univariate tests indicated that: obese women had significantly lower Big IGF-II (Mean (*M*) = .95, Standard Deviation (*SD*) = .29) than the normal weight women (*M* = 1.06, *SD* = .29), *p* = .028; the overweight women had higher Mature IGF-II (*M* = .96, *SD* = .22) than the normal weight women (*M* = .88, *SD* = .17), *p* = .021; but that Pro IGF-II for the overweight women (*M* = .96, *SD* = .22) and normal weight women (*M* = 1.00, *SD* = .21) were not significantly different, *p* = .269. To further investigate these differences, we assessed if the overweight BED, overweight non-BED, and normal weight women differed on the IGF-II variables. Table 2 shows the means, standard deviations by group. The multivariate test was significant, *p*<.001, and the univariate tests were significant for the Big and Mature IGF-II. Tukey's post hoc comparisons indicated that the overweight women without BED had significantly lower Big IGF-II than the normal weight women, *p*<.05. Tukey's test also showed that the overweight women with BED had higher Mature IGF-II than the normal weight women, *p*<.05.

**Table 2:** Means, standard deviations (*SD*), univariate p-values, and Tukey's post-hoc paired comparisons results for each IGF-II variable by group.

IGF-II	BED (*n* = 97)	Overweight (*n* = 53)	Normal Weight (*n* = 50)	*Univariatep*	Tukey's Post Hoc Tests
Mean PRO (*SD*)	0.98 (0.25)	0.93 (0.18)	1.00 (0.21)	.219	n.s.
Mean BIG (*SD*)	0.97 (0.31)	0.90(0.25)	1.06 (0.29)	.033	OW<NW
Mean Mature (*SD*)	0.98 (0.23)	0.92 (0.19)	0.88 (0.17)	.020	BED>NW

BED = overweight binge eating disorder; OW = overweight group; NW = normal weight group.

Next, we assessed if IGF-II variables changed from pre- to post- to six months post-group psychological treatment for the women with BED after controlling for baseline scores and group psychological treatment condition. Table 3 shows the means and standard deviations for each IGF-II variable at each assessment period for the sample that provided data. Linear slope parameters from the MLM indicated that neither Pro IGF-II (β_10_ = .004, *t* = 0.235, *p* = .727) nor Mature IGF-II (β_10_ = −.020, *t* = −1.95, *p* = .054) changed across assessment periods. However, Big IGF-II did show a significant decreasing slope from pre- to post- to six months post-group psychological treatment (β_10_ = −.039, *t* = −2.71, *p* = .008). We then assessed if change in BMI, a time varying covariate in the MLM, was associated with change in IGF-II variables. Change in BMI was not associated with change in any of the IGF-II variables (*p*>.05).

**Table 3:** Means and standard deviations (SD) of each IGF-II variable across three group psychological treatment assessment periods for women with binge eating disorder.

IGF-II	Pre (*n* = 97)	Post (*n* = 64)	6 Months (*n* = 56)
Mean PRO (*SD*)	0.98 (0.25)	0.99 (0.18)	1.00 (0.20)
Mean BIG (*SD*)	0.97 (0.31)	1.02 (0.45)	0.90 (0.31)
Mean Mature (*SD*)	0.98 (0.24)	0.98 (0.20)	0.94 (0.22)

## Discussion

Using a well characterized prospective cohort we demonstrated for the first time that women suffering from BED display significant alterations in circulating concentrations of various IGF-II isoforms, independently of their BMI or of changes in this index. In particular, overweight participants with BED displayed increased circulating levels of mature IGF-II compared to normal weight controls whereas overweight women without BED had decreased serum levels of Big IGF-II compared to the same controls. This suggested a specific association between BED and altered IGF-II processing, favoring the release of mature IGF-II.

The pattern of IGF-II isoforms specifically observed in our BED population may reflect abnormal processing by PC4 (pro protein convertase 4) which we have previously shown is responsible for the final maturation of IGF-II from the pro and Big forms [9]. Thus, overexpression of PC4 in BED subjects could lead to such an aberration in IGF-II isoforms, although this requires further study particularly since PC4 is known to be expressed only in reproductive tissues and has never been shown to play a role in body weight regulation.

Alternately, it is possible that mature IGF-II itself may be “nutritionally regulated” as suggested by the literature describing a correlation between obesity and elevated levels [7]. Our own findings support this possibility by revealing a positive correlation between BMI and levels of mature IGF-II, an isoform which in other tissues (placenta) has been found to be the most biologically active [9]. Furthermore, IGF-II and its receptors are expressed in the hypothalamus, particularly in regions involved in the regulation of food intake such as the ventromedial and paraventricular nuclei [26]. In addition, administration of insulin has been shown to promote IGF-II expression in these areas of the hypothalamus [27]. Taken together these data suggest a neuroendocrine function of IGF-II in the control of food intake, although the role of specific isoforms remains unclear.

One prior study has examined the influence of obesity and weight loss on pro and mature IGF-II [12]. In a cohort of 34 obese individuals, pro-IGF II (1–156) was found to be decreased compared to normal weight individuals. Since obesity was associated with an upregulation of mature IGF-II, authors speculated that abnormal enzymatic processing of IGF-II could be responsible for the altered levels of IGF-II isoforms observed. However, the lack of correlation between pro-IGF-II and mature IGF-II levels found in other studies fail to support this possibility [28]. Investigators concluded that pro-IGF-II was likely nutritionally regulated and this, independently of IGF-II. Our data contrasts with these results on pro-IGF-II as levels of this isoform were not correlated with BMI and not altered by obesity. These contradictory results may be explained by the very different methodologies used in the measurement of specific isoforms. We have opted for a Western approach which allows us to identify clearly individual isoforms. However, the pro-IGF II study described above relied on an in house sandwich assay which carried a 20% cross reactivity between pro-IGF II and mature IGF-II.

Although we did not find altered pro-IGF-II (1–156) levels in our BED population, the increased mature IGF-II levels noted could play a role in the neurobiology of this disease. IGF-II is known to be secreted peripherally and centrally and to cross the blood brain barrier. If, as mentioned above, mature IGF-II is most biologically active in the hypothalamus as it is in other tissues, its influence on feeding behavior could be physiologically relevant. In healthy individuals, food intake results in increased circulating insulin which in turn is well known to increase IGF-II concentrations. Insulin like growth factor II, acting on the hypothalamus, has been shown to decrease the release of neuropeptide Y (NPY) from the paraventricular nucleus [29]. NPY is one of the most potent regulators of feeding behavior. Injections of NPY lead to increased feeding whereas starvation results in its decreased expression [30], [31]. As such, the IGF-II mediated decrease in NPY leads to a cessation of food intake. Our BED subjects have consistently shown elevations in the functional mature form of IGF-II, likely as a result of the continuous stimulus of insulin resulting from their binge eating. Consequently, a decrease in NPY should be expected thereby leading to cessation of food intake. However, this fails to occur in BED individuals and current data suggest that levels of NPY might indeed be increased in individuals with this disorder. For instance, a rat model of overeating created by gastric overdistention revealed that this behavior became gradually associated with higher fasting NPY concentrations [32]. Whether this is also the case in human BED has not been investigated. Taken together, these data suggest that NPY is resistant to the insulin mediated mature IGF-II increase occurring in this eating disorder. As anticipated decreases in NPY do not occur, hyperphagia persists along with further elevations in IGF-II concentrations in an attempt to regulate NPY [33]. Since levels of mature IGF-II were not elevated in obese individuals without BED, this suggests that this particular isoform may be more relevant in the neuropathology of BED. Indeed obese women without BED displayed increased Big IGF-II, a potentially less biologically potent isoform.

Finally, it is also possible that changes in IGF-II levels are mediated by alterations in serotonin concentrations. Individuals with BED often have associated co-morbidities such as depression and insulin resistance [4], [5]. Both these conditions have been found to be associated with decreased circulating levels of serotonin, which itself has been inversely correlated with IGF-II expression [14]. This could partly explain the rise in mature IGF-II seen in overweight women suffering from BED.

Our study design allowed us to also investigate whether changes in IGF-II isoform concentrations were seen following group psychological treatment of BED. Although pro and Mature IGF-II remained unchanged, levels of Big IGF-II decreased significantly following group therapy which itself was associated with decreased binging and depression, but not with a significant weight difference [16]. Therefore, this decrease in levels of Big IGF-II cannot be explained by change in body weight. This is an intriguing finding which may reflect an early improvement in the act of binging itself. Indeed in our study, overweight women without BED showed lower levels of Big IGF-II compared to healthy controls. Therefore, our “treated BED population” might start to display a profile more similar to that of overweight non BED women. It is also possible that these changes may be related to improvements in depression associated with the group psychological treatment which, mediated by rising serotonin, might contribute to decreased IGF expression. It is unclear why mature IGF-II levels did not change in a similar fashion. Whether this is related to sensitivity to serotonin, or other post translational factors should be examined. Whether levels of Big IGF-II could then be used therapeutically to evaluate binging activity and response to treatment also remains to be investigated. The results provide preliminary evidence that BIG IGF-II is sensitive to change due to group psychological treatment.

Our study is a first attempt to examine the IGF-II system in BED. Its strengths include the use of a Western to assess IGF-II isoforms, allowing differentiation of these. We minimized inter and intra-observer variability that is a typical weakness of the Western by having all samples assayed at the same time and by a single investigator who was blinded to the study groups. However, small errors, for example in loading, remain a possibility. We took into account other variables which could potentially affect IGF-II circulating levels, notably cancer, pregnancy, diabetes (excluded) and menopausal status. Limitations include the fact that the Western approach although ideal to discriminate between isoforms, is not applicable to large samples. Finally another consideration is that because BED usually co-exists with other conditions such as depression, it is difficult to account for their potential influence on IGF-II regulation. We believe that BED and depression may in fact be interrelated and likely part of the same pathophysiology. As such, controlling for depression would be inappropriate.

In conclusion, we have shown that overweight women with BED display abnormal levels of circulating IGF-II isoforms. In particular BED is characterized by elevated mature IGF-II, an isoform previously shown to carry significant bioactivity. This finding is not related to BMI or to changes in body weight. We suggest that abnormalities in IGF-II processing may be involved in the neurobiology of BED. Group psychological treatment may impact levels of BIG IGF-II that is perhaps associated with change in depression and serotonin. Our findings need to be replicated in a larger population with an attempt to further unravel the underlying mechanisms involved by examining potential targets of IGF-II that are involved in satiety control.
