# Correction: Characterization of IGF-II Isoforms in Binge Eating Disorder and Its Group Psychological Treatment

**DOI:** 10.1371/annotation/bb067823-48b8-4573-907e-224cb73917a2

**Published:** 2014-01-15

**Authors:** Giorgio Tasca, Julien Yockell Lelievre, Qing Qiu, Kerri Ritchie, Julian Little, Anne Trinneer, Ann Barber, Livia Chyurlia, Hany Bissada, Andreé Gruslin

In the byline, the name of the fifth author is incorrect. The correct name is: Julian Little.

